# ^18^F-labeled Pyrazolo[1,5-a]pyrimidine Derivatives: Synthesis from 2,4-Dinitrobenzamide and Tosylate Precursors and Comparative Biological Evaluation for Tumor Imaging with Positron Emission Tomography

**DOI:** 10.3390/molecules17043774

**Published:** 2012-03-27

**Authors:** Jingli Xu, Hang Liu, Guixia Li, Yong He, Rui Ding, Xiao Wang, Man Feng, Shuting Zhang, Yurong Chen, Shilei Li, Mingxia Zhao, Yingruo Li, Chuanmin Qi, Yonghong Dang

**Affiliations:** 1 Key Laboratory of Radiopharmaceuticals (Beijing Normal University), Ministry of Education, College of Chemistry, Beijing Normal University, Beijing 100875, China; 2 Department of Nuclear Medicine, PUMC Hospital, Beijing 100730, China

**Keywords:** ^18^F-labeled, pyrazolo[1,5-a]pyrimidine derivatives, 2,4-dinitrobenzamide and tosylate precursors, PET imaging agents, tumor detection

## Abstract

We previously reported ^18^F-labeled pyrazolo[1,5-a]pyrimidine derivatives: 7-(2-[^18^F]fluoroethylamino)-5-methylpyrazolo[1,5-a]pyrimidine-3-carbonitrile ([^18^F]**1**) and *N*-(2-(3-cyano-5-methylpyrazolo[1,5-a]pyrimidin-7-ylamino)ethyl)-2-[^18^F]fluoro-4-nitro- benzamide ([^18^F]**2**). Preliminary biodistribution experiments of both compounds showed s slow clearance rate from excretory tissues which warranted further investigation for tumor imaging with PET. Here we modified [^18^F]**1** and [^18^F]**2** by introducing polar groups such as ester, hydroxyl and carboxyl and developed three additional ^18^F-18 labeled pyrazolo[1,5-a] pyrimidine derivatives: (3-Cyano-7-(2-[^18^F]fluoroethylamino)pyrazolo[1,5-a]-pyrimidin-5- yl)methyl acetate ([^18^F]**3**), 7-(2-[^18^F]fluoroethylamino)-5-(hydroxymethyl)pyrazolo[1,5-a]- pyrimidine-3-carbonitrile ([^18^F]**4**) and (*S*)-6-(3-cyano-5-methylpyrazolo[1,5-a]pyrimidin-7- ylamino)-2-(2-[^18^F]fluoro-4-nitrobenzamido)hexanoic acid ([^18^F]**5**). The radiolabeled probes were synthesized by nucleophilic substitution of the corresponding tosylate and nitro precursors with ^18^F-fluoride. *In Vitro* studies showed higher uptake of [^18^F]**3** and [^18^F]**4** than that of [^18^F]**5** by S180 tumor cells. *In Vivo* biodistribution studies in mice bearing S180 tumors showed that the uptake of both [^18^F]**3** and [^18^F]**4** in tumors displayed an increasing trend while the uptake of [^18^F]**5** in tumor decreased through the course of the 120 min study. This significant difference in tumor uptake was also found between [^18^F]**1** and [^18^F]**2**. Thus, we compared the biological behavior of the five tracers and reported the tumor uptake kinetic differences between 2-[^18^F]fluoroethylamino- and 2-[^18^F]fluoro-4-nitro- benzamidopyrazolo[1,5-a] pyrimidine derivatives.

## 1. Introduction

2-[^18^F]Fluoro-2-deoxyglucose (^18^F-FDG) positron emission tomography (PET) is generally accepted as a routine method in clinical diagnosis and evaluation of cancer. However, this tracer has inherent drawbacks. For example, its high accumulation in inflammed and infected tissues can lead to false-positive results and its low uptake in tumors that are growing slowly can cause false-negative results [1,2]. Consequently, there is a need to develop new PET tumor-imaging agents. Pyrazolo- pyrimidines have multiple pharmacological activities including hypnotic [3], anti-inflammatory [4], anti-tumor [5,6,7,8,9], antimycobacterial [10] and anti-viral properties [11,12]. Pyrazolo[1,5-a]pyrimidine and its derivatives have attracted broad interest over the years due to their structural resemblance to purine [13]. They were found to have antitrypanosomal [14] and antischistosomal activity [15], and most importantly, anti-tumor activity [16,17,18,19]. Furthermore, many pyrazolo[1,5-a]pyrimidine derivatives were proved to block proliferation of various cancer cell lines [17,20,21]. As a result, pyrazolo[1,5-a]pyrimidine has been widely applied as an important pharmacophore or building block in anti-tumor drug design, which has motivated further study on the physiological and biological properties of this kind of compounds, including *in Vitro* stability, *in Vivo* distribution, metabolism and elimination. 

In a previous paper, we reported our initial efforts to design and label the pyrazolo[1,5-a]pyrimidine pharmacophore from the corresponding tosylate and nitro precursors to get compounds [^18^F]**1** and [^18^F]**2** with the aim of developing new radiotracers for tumor imaging by means of PET. The studies of *in Vitro* cell uptake and *in Vivo* biodistribution demonstrated that both tracers had more accumulation in the tumors than L-[^18^F]FET and [^18^F]FDG. Unfortunately, their low tumor-to-non target ratios limited their application in PET tumor imaging. These data suggested that our important design consideration for labeling pyrazolo[1,5-a]pyrimidine pharmacophore with ^18^F-fluoride is to maintain the high concentration of radioactivity in the tumor and improve the clearance rate from other organs. What’s more, in our previous work we noted that [^18^F]**1 **and [^18^F]**2**, which had similar lipophilicity, showed quite different biodistribution.

**Figure 1:**
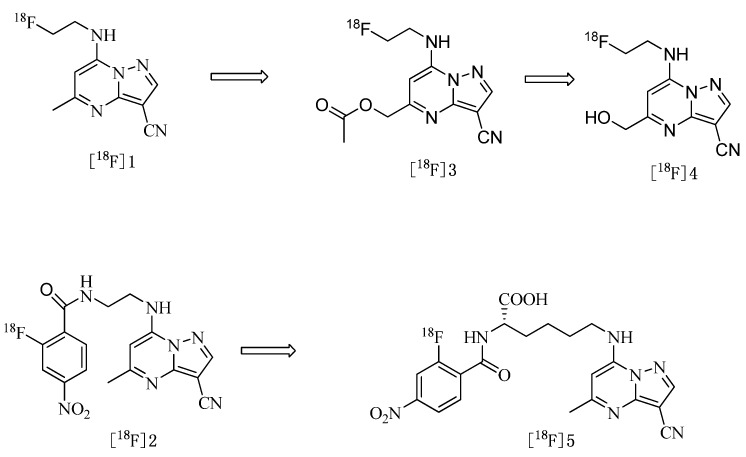
Structures of ^18^F labeled pyrazolo[1,5-a]pyrimidine derivatives.

The objective of this study was to lower the lipophilicity of [^18^F]**1** and [^18^F]**2** by the introduction of ester, hydroxyl and carboxyl groups, which have been extensively used to modify the lipophilicity properties of other tracers and therefore their biodistribution [22] to produce the compounds with potential applicability in PET imaging. We thus designed [^18^F]**3**, [^18^F]**4** and [^18^F]**5** (Figure 1) and intended to find some relationship among the structure, the physical property (e.g., lipophilicity) and the biological property (e.g., accumulation in tumor). In addition, we attempted to label pyrazolo[1,5-a]pyrimidine pharmacophore with ^18^F-fluoride employing tosylate and nitro compounds as precursors and identify the influences of two kinds of ^18^F labeled pyrazolo[1,5-a]pyrimidine pharmacophore on the tumor uptake.

In view of the above mentioned facts we successively synthesized three novel ^18^F labeled pyrazolo[1,5-a]pyrimidine derivatives: [^18^F]**3**, [^18^F]**4** and [^18^F]**5** and investigated their radiopharmacological characterization: Cellular uptake and biological activities in mice bearing S180 tumor (sarcoma S180 has been widely used to evaluate the antitumor activities of drugs due to its growth advantage [23,24,25,26]). Furthermore, we compared the cellular uptake of [^18^F]**3**, [^18^F]**4** and [^18^F]**5** with that of [^18^F]**FDG**, biodistribution data of [^18^F]**3**, [^18^F]**4** and [^18^F]**5** with those of [^18^F]**1** and [^18^F]**2**. The data of [^18^F]**FDG**, [^18^F]**1** and [^18^F]**2** were obtained using the previously described [27].

## 2. Results and discussion

### 2.1. Chemistry

**Scheme 1:**
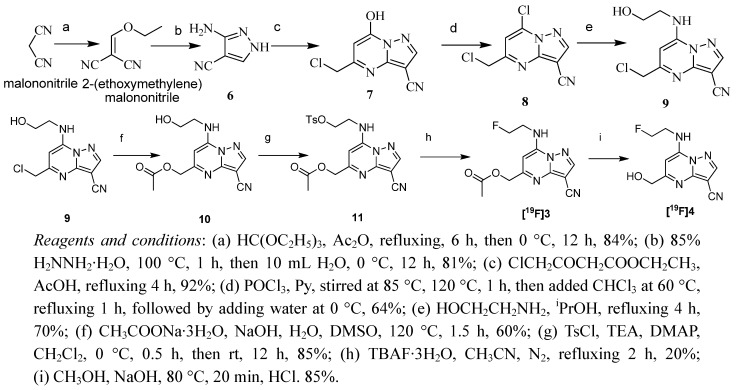
Synthesis of [^19^F]**3** and [^19^F]**4**.

**Scheme 2:**
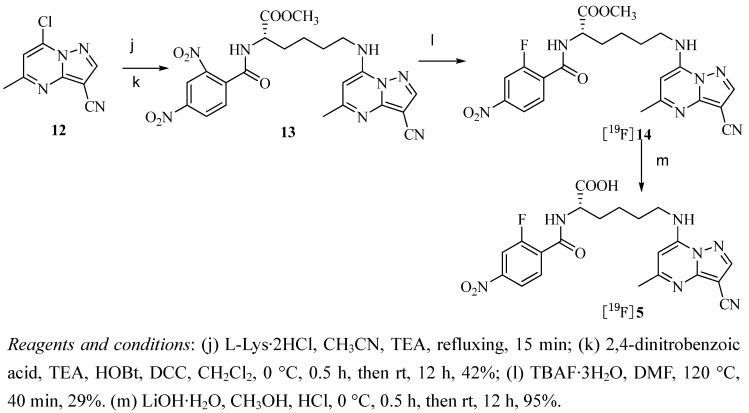
Synthesis of [^19^F]**5**.

The synthesis route of [^19^F]**3** and [^19^F]**4**, shown in Scheme 1, is a modification of the synthesis of [^19^F]**1** described by us previously [27]. Treatment of **9** with sodium acetate in a basic mixture of H_2_O and DMSO solution generated the desired product **10**. Subsequent reaction with *p*-toluenesulfonyl chloride (1.5 eq.) in dry dichloromethane containing triethylamine (1.5 eq.) and a catalytic amount of 4-(N,N-dimethylamino)pyridine (DMAP) at room temperature overnight gave the target precursor **11**. ^19^F substituted [^19^F]**3** was obtained from precursor **11** using TBAF·3H_2_O in dry acetonitrile at 75 °C under nitrogen atmosphere for 2 h. Preparation of [^19^F]**4** was accomplished by hydrolysis of [^19^F]**3** with NaOH in MeOH, subsequent acidification with 1 N HCl and purification by recrystallization. The synthesis procedure of [^19^F]**5**, as illustrated in Scheme 2, is the modification of the route to [^19^F]**2** described by us previously [27]. The 2,4-dinitrobenzaldehyde group was linked through L-lysine to the pyrazolo[1,5-a]pyrimidine core. L-Lysine methyl ester hydrochloride was reacted with **12** in the presence of triethylamine to give the lysine pyrazolo[1,5-a]pyrimidine and coupled to 2,4-dinitrobenzoic acid using HOBt and DCC to provide the precursor **13**. Finally [^19^F]**5** was obtained by hydrolysis of [^19^F]**14** with LiOH·2H_2_O in MeOH, subsequent acidification with 1 N HCl and purification by recrystallization. 

### 2.2. Radiochemistry

**Scheme 3:**
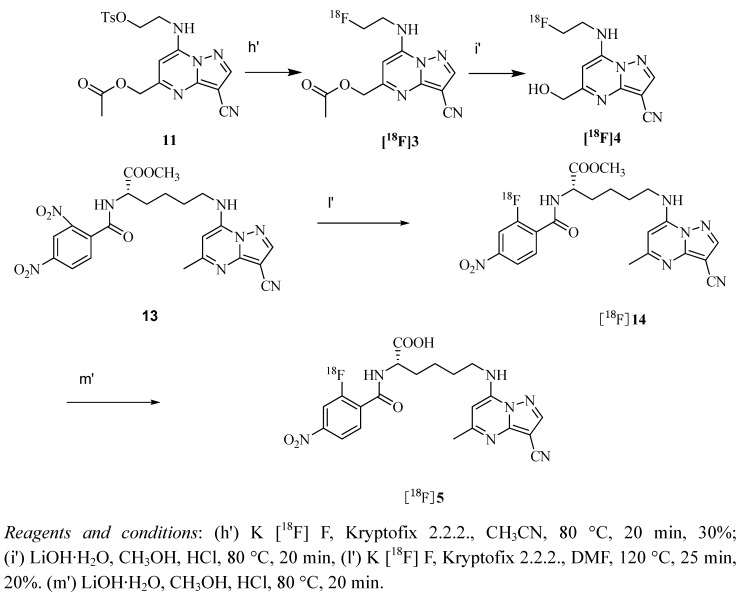
Radiochemical synthesis of [^18^F]**3**, [^18^F]**4** and [^18^F]**5**.

The radiosynthesis from 2,4-dinitrobenzamide and tosylate precursors followed the conditions as shown in Scheme 3. The synthesis of all the three radiotracers required about 2 h with overall radiochemical yields of 20–30% non decay-corrected, radiochemical purity >98% and specific activity between 31–40 GBq/μmol. The identity of the radiolabeled products was confirmed by comparing the retention time of radioactive product with that of ^19^F-compound by HPLC. The retention time of [^18^F]**3**, [^18^F]**4** and [^18^F]**5** were 11.3 min, 6.6 min and 2.3 min, respectively, which matched well with [^19^F]**3** (11.0 min), [^19^F]**4** (6.3 min) and [^19^F]**5** (2.1 min) within admissible error. All dry radiolabeled products then formulated in physiological phosphate buffer solution (pH = 7.4).

### 2.3. Measurement of Partition Coefficient

The partition coefficient (logP) values of [^18^F]**3**, [^18^F]**4** and [^18^F]**5** were −0.04, −0.09 and −1.78 respectively, much lower than those of [^18^F]**1**(0.68) and [^18^F]**2**(0.69), suggesting those radiotracers were hydrophilic. Clearly, the introduction of ester, hydroxyl and carboxyl groups at the C-5 or C-7 positions of the pyrazolo[1,5-a]pyrimidine core structure significantly decreased the lipophilicity of [^18^F]**1** and [^18^F]**2**.

### 2.4. Stability of [^18^F]3, [^18^F]4 and [^18^F]5

The Radio-HPLC showed that all the three radiotracers were stable in both phosphate-buffered saline (pH 7.4) and murine plasma at 37 °C after 2 h.

### 2.5. Cellular Accumulation of [^18^F]3, [^18^F]4 and [^18^F]5

**Figure 2:**
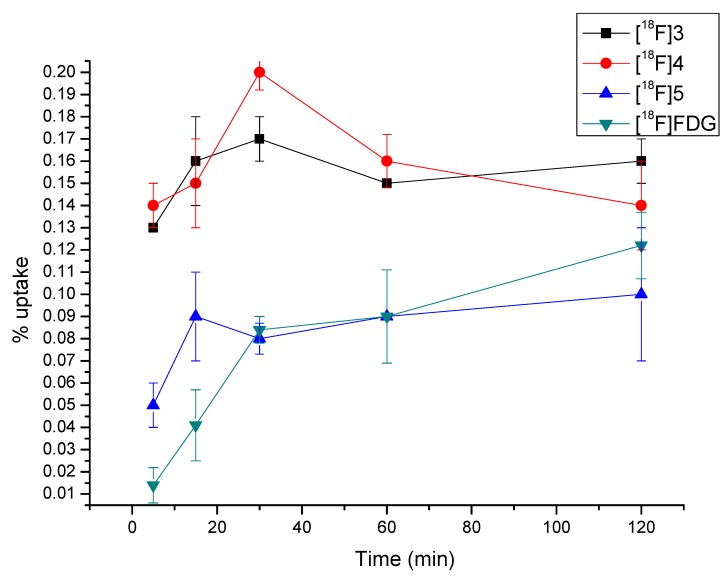
Uptake kinetics of [^18^F]**3**, [^18^F]**4**, [^18^F]**5** and [^18^F]FDG into S180 tumor cells at 37 °C (pH = 7.4). Data are expressed as % uptake (mean with S.D. n = 5 each). *P *< 0.05.

Cell uptake [27] values of [^18^F]**3**, [^18^F]**4** and [^18^F]**5** at 37 °C over incubation time of 5, 15, 30, 60 and 120 min are shown in Figure 2. [^18^F]**3** exhibited rapid accumulation in S180 tumor cells, reached the peak value of 0.17 ± 0.1% at 30 min and maintained the high uptake value at 2 h (0.16 ± 0.1%). A similar cell uptake pattern was observed for [^18^F]**4**.

**Figure 3:**
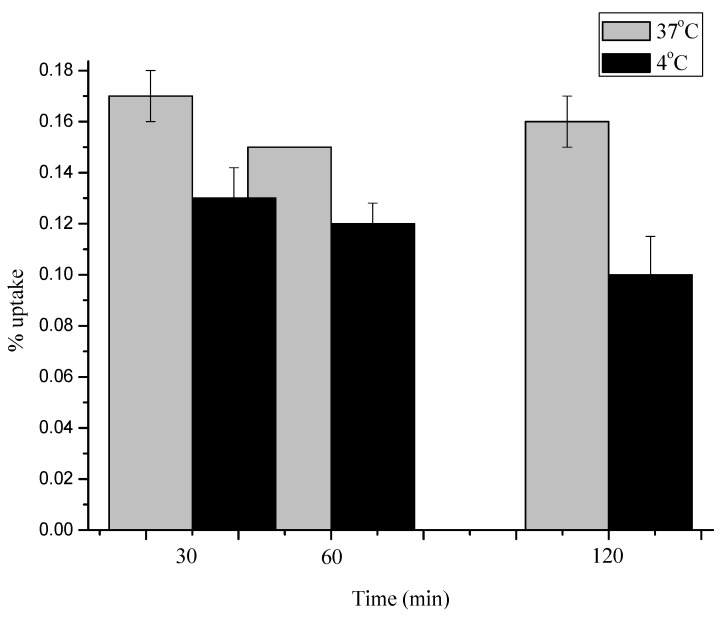
Comparison of uptake kinetics of [^18^F]**3** into S180 tumor cells at 37 °C and 4 °C (pH = 7.4). Data are expressed as % uptake (mean with S.D. n = 5 each). *P *< 0.05.

The peak value occurred at 30 min (0.2 ± 0.08%) and slightly decreased at 2 h (0.14 ± 0.2%). The uptake of [^18^F]FDG was rapid for the first 30 minutes, followed by a nearly steady state from 30 minutes onward. Compared to [^18^F]FDG, the cell uptake of [^18^F]**3** and [^18^F]**4** was much higher at all the selected time points. Uptake of [^18^F]**5** was significantly lower than [^18^F]**3** and [^18^F]**4**, but similar to [^18^F]FDG at all time points. The results were consistent with *ex vivo* biodistribution studies. Moreover, uptake of all the three radiotracers showed only slight temperature dependence in our study (data of [^18^F]**3** shown in Figure 3, data of [^18^F]**4** and [^18^F]**5** not shown). As no significant difference between the *in Vitro* uptake kinetics of [^18^F]3, [^18^F]4 or [^18^F]5 at 4 °C and at 37 °C could be observed, it can be concluded that said compounds enter the cell by passive diffusion and not by an active transport mechanism, in agreement with *ex vivo* biodistribution data.

### 2.6. Biodistribution of [^18^F]3, [^18^F]4 and [^18^F]5 in S180 Bearing Mice

The biodistribution data of [^18^F]**3**, [^18^F]**4** and [^18^F]**5** were summarized in Table 1, Table 2 and Table 3, respectively. Figure 3 showed the direct comparison of tumor uptake (%ID/g) among [^18^F]**1**, [^18^F]**2**, [^18^F]**3**, [^18^F]**4** and [^18^F]**5** in the same animal model. The biodistribution data of [^18^F]**1** and [^18^F]**2** were obtained from our previous report [27].

**Table 1:** Biodistribution of [^18^F]**3** in mice bearing S 180 tumor. Expressed as % injected dose per gram (% ID/g ± SD), n = 4.

Organs	Time (min)				
	5	15	30	60	120
Heart	6.97 ± 0.47	5.08 ± 1.50	4.78 ± 0.36	3.63 ± 0.65	2.50 ± 0.67
Liver	10.3 ± 0.44	6.03 ± 0.48	4.72 ± 0.81	2.97 ± 0.41	1.82 ± 0.079
Spleen	6.92 ± 0.95	4.02 ± 0.90	3.14 ± 0.096	3.09 ± 0.69	1.66 ± 0.080
Lung	6.21 ± 1.12	4.96 ± 1.08	4.02 ± 0.48	3.38 ± 0.38	1.88 ± 0.32
Kidney	9.36 ± 0.25	4.97 ± 0.66	4.26 ± 0.29	2.94 ± 0.43	2.08 ± 0.26
Brain	2.38 ± 0.17	2.30 ± 0.62	2.27 ± 0.69	2.18 ± 0.29	1.47 ± 0.14
Muscle	6.64 ± 0.91	4.02 ± 1.03	3.79 ± 0.31	3.59 ± 0.43	2.12 ± 0.69
Blood	6.09 ± 0.25	4.46 ± 0.18	4.43 ± 0.30	3.78 ± 0.56	2.59 ± 0.14
Tumor	3.02 ± 0.36	3.45 ± 0.46	5.03 ± 0.68	3.96 ± 1.10	2.94 ± 0.27
Tumor/Brain	1.27	1.50	2.21	1.82	2.00
Tumor/Muscle	0.45	0.86	1.33	1.10	1.39
Tumor/Blood	0.49	0.77	1.14	1.05	1.14

**Table 2:** Biodistribution of [^18^F]**4** in mice bearing S 180 tumor. Expressed as % injected dose per gram (% ID/g ± SD), n = 4.

Organs	Time (min)				
	5	15	30	60	120
Heart	6.67 ± 0.92	4.20 ± 1.0	5.07 ± 0.96	4.09 ± 0.59	3.61 ± 1.1
Liver	10.7 ± 0.65	6.05 ± 0.56	4.61 ± 1.0	3.07 ± 0.35	2.32 ± 0.18
Spleen	6.97 ± 0.34	3.87 ± 0.13	4.33 ± 1.3	2.99 ± 0.66	2.34 ± 0.69
Lung	6.33 ± 0.87	4.06 ± 0.41	4.07 ± 0.18	3.24 ± 0.73	2.58 ± 0.30
Kidney	11.1 ± 1.4	5.86 ± 0.14	4.2 ± 0.52	3.03 ± 0.55	2.58 ± 0.48
Brain	1.75 ± 0.18	2.33 ± 0.26	2.95 ± 0.56	2.26 ± 0.49	1.6 ± 0.16
Muscle	6.48 ± 1.0	3.75 ± 0.32	4.19 ± 0.65	3.28 ± 0.71	1.54 ± 0.39
Blood	6.33 ± 0.45	4.91 ± 0.46	4.57 ± 0.59	3.64 ± 0.40	3.30 ± 0.22
Tumor	1.91 ± 1.2	3.84 ± 0.46	4.41 ± 1.3	4.53 ± 0.90	3.75 ± 0.21
Tumor/Brain	1.09	1.65	1.5	2.01	2.35
Tumor/Muscle	0.29	1.03	1.05	1.38	2.44
Tumor/Blood	0.30	0.78	0.96	1.25	1.14

**Table 3:** Biodistribution of [^18^F]**5** in mice bearing S 180 tumor. Expressed as % injected dose per gram (% ID/g ± SD), n = 4.

Organs	Time (min)				
	5	15	30	60	120
Heart	3.02 ± 0.51	1.19 ± 0.21	0.97 ± 0.19	0.42 ± 0.07	0.52 ± 0.05
Liver	10.39 ± 1.86	2.90 ± 0.86	2.29 ± 0.38	1.79 ± 0.38	1.63 ± 0.22
Spleen	3.10 ± 0.12	2.76 ± 0.11	0.54 ± 0.07	0.55 ± 0.19	0.53 ± 0.02
Lung	16.34 ± 1.80	15.13 ±1.57	6.35 ± 1.28	2.45 ± 0.55	4.09 ± 0.19
Kidney	12.70 ± 0.90	3.71 ± 0.69	2.21 ± 0.29	0.94 ± 0.28	0.58 ± 0.01
Brain	0.22 ± 0.00	0.14 ± 0.00	0.15 ± 0.02	0.15 ± 0.00	0.14 ± 0.00
Muscle	2.15 ± 0.66	1.07 ± 0.35	0.49 ± 0.09	0.54 ± 0.00	0.18 ± 0.04
Blood	6.68 ± 0.05	2.24 ± 0.04	0.95 ± 0.10	0.45 ± 0.09	0.35 ± 0.02
Tumor	2.55 ± 0.42	1.75 ± 0.54	1.30 ± 0.15	0.71 ± 0.11	0.83 ± 0.17
Tumor/Brain	11.59	12.5	8.67	4.73	5.93
Tumor/Muscle	1.19	1.64	2.65	1.31	4.61
Tumor/Blood	0.38	0.78	1.37	1.58	2.37

In general, as shown in Table 1 and Table 2, [^18^F]**3** and [^18^F]**4** accumulated to high levels in the tumor, 5.03 ± 0.68 and 4.41 ± 1.3 %ID/g at 30 min post-injection, respectively, and stayed at similarly high levels 3.96 ± 1.10 and 4.53 ± 0.90 %ID/g at 60 min post-injection, 2.94 ± 0.27 and 3.75 ± 0.21 %ID/g at 120 min post-injection, respectively, demonstrating persistent retention over time. Meanwhile, they also showed moderate clearance from other organs and tissues, resulting in tumor/brain, tumor/muscle and tumor/blood ratios of 2.00, 1.39 and 1.14 for [^18^F]**3**, 2.35, 2.44 and 1.14 for [^18^F]4 at 120 min post-injection. [^18^F]**5**, which contains a carboxyl group, exhibited high liver and kidney uptake, 10.39 ± 1.86 and 12.70 ± 0.90 %ID/g at 5 min post-injection, respectively, and rapid clearance of 1.63 ± 0.22 and 0.58 ± 0.01 %ID/g at 120 min post-injection, respectively. Surprisingly, The uptake of [^18^F]**5** in lung was relatively higher than that in other tissues at all time points studied. This phenomenon might result from a passive process of the radiotracers in lung. Furthermore, this tracer displayed 2.55 ± 0.42 %ID/g in tumor at the earliest (5 min) and then it was gradually reducing over the 2 h time period of the study, which indicated that [^18^F]**5** had insignificant retention in tumor. In addition, very low brain accumulation afforded high tumor/brain ratios: 11.59, 12.5, 8.67, 4.73 and 5.93. The ratios of tumor to other tissues were over 1 except for the ratios of tumor to liver (0.5) and tumor to kidney (0.02), on the other hand, the tumor/muscle and tumor/blood ratios reached their peak value of 4.61 and 2.37 respectively at 120 min post-injection. 

**Figure 4:**
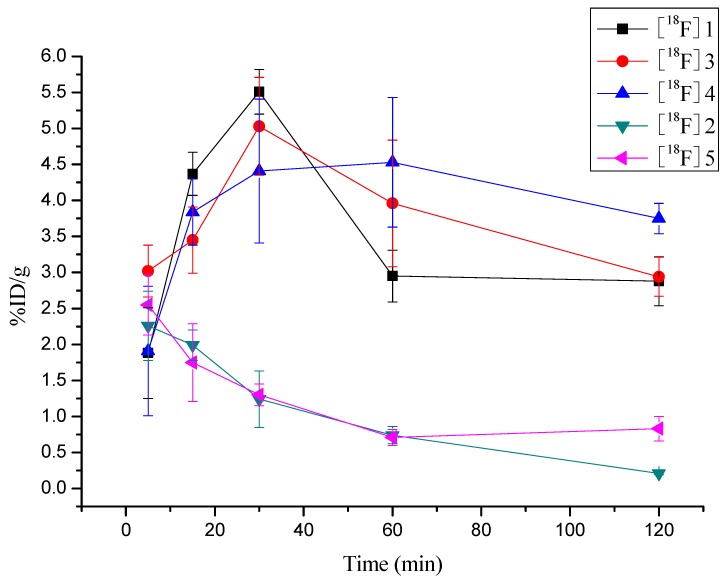
Comparison of tumor uptake rates of [^18^F]**1**, [^18^F]**3** and [^18^F]**4** with those of [^18^F]**2** and [^18^F]**5** in the same animal model bearing S180 tumor.

Biodistribution studies revealed generally similar uptake patterns of [^18^F]**1**, [^18^F]**3** and [^18^F]**4** in most organs and the tumor. High initial activity accumulations but slow reduction in the excretory tissues for all the three radiotracers were observed, which may be the result of the relatively slow clearance from blood. However, all the three radiotracers displayed a remarkable increasing uptake in tumor during *in Vivo* animal studies as shown in Figure 4. [^18^F]**1** and [^18^F]**3** reached their peak tumor uptake at 30 min post-injection (5.51 ± 0.31 %ID/g for [^18^F]**1** and 5.03 ± 0.68 %ID/g for [^18^F]**3**) while [^18^F]**4** did at 60 min post-injection (4.53 ± 0.90 %ID/g for [^18^F]**4**). After the peak, the uptake of all the three radiotracers started to decline. After 120 minutes post injection, the radioactivity was about half of the peak value. They were 2.88 ± 0.34 %ID/g for [^18^F]**1**, 2.94 ± 0.27 %ID/g for [^18^F]**3** and 3.75 ± 0.21 %ID/g for [^18^F]**4 ** respectively. These data indicated that all the three tracers remained in tumor for a relatively long time. What’s more, the ratios of tumor to brain, muscle and blood for all three tracers at each selected time points were close. These findings demonstrated that the decreased lipophilicity caused by introduction of ester and hydroxyl groups at the C-5 position of the pyrazolo[1,5-a]pyrimidine core structure did not affect the *in Vivo* biological behavior of these compounds.

On the other hand, [^18^F]**5** showed similar distribution patterns as [^18^F]**2** in most organs, including the tumor. For both radiotracers, peak uptake in all organs and tissues occurred at the initial time and then they washed out swiftly from these organs and tissues as time elapsed. Additionally, radioactivity concentrations in tumor was 2.26 ± 0.48 %ID/g for [^18^F]**2** and 2.55 ± 0.42 %ID/g for [^18^F]**5** at 5 min p.i. and continually decreased to 0.21 ± 0.02 %ID/g for [^18^F]**2** and 0.83 ± 0.17 %ID/g for [^18^F]**5** at 60 min p.i., indicating temporary retention in tumor. Due to relatively low uptake into brain, both radiotracers afforded high tumor/brain ratios at each selected time point. The tumor/blood ratios of both radiotracers were close at each selected time points while the tumor/muscle ratios of [^18^F]**5** were higher than those of [^18^F]**2** at 5, 15, 30 and 120 min. These data confirmed that the decreased lipophilicity caused by introduction of the carboxyl group at the C-7 position of the pyrazolo[1,5-a]pyrimidine core structure gave rise to insignificant biological behavior of these compounds *in Vivo*.

Overall, the total yields of the radiolabeled products obtained from 2,4-dinitrobenzamide and tosylate precursors were comparable, but statistically significant differences in tumor uptakes were observed: all tracers [^18^F]**1**, [^18^F]**3** and [^18^F]**4** had higher uptake and longer retention in tumor than those of both tracers [^18^F]**2** and [^18^F]**5**. These differences are noteworthy, as they suggest that the 2-[^18^F]fluoro-4-nitrobenzamide group on position 7 seems to have negative effect on the tumor uptake kinetic when compared to a 2-[^18^F]fluoroethylamino group in this position. The findings could not be explained simply by the differential lipophilicity. [^18^F]**1**, [^18^F]**3** and [^18^F]**4** showed the similar biological behavior including the tumor uptake kinetic despite they possess the distinct lipophilicity. [^18^F]**2**(0.68) and [^18^F]**5**(−1.78) exhibited the same findings as [^18^F]**1**, [^18^F]**3** and [^18^F]**4**. The different metabolic fate of these compounds could be the possible answer for this finding. By comparing the parallel biodistribution of 2-[^18^F]fluoroethylaminopyrazolo[1,5-a]pyrimidine derivatives and 2-[^18^F]- fluoro-4-nitrobenzamideopyrazolo[1,5-a]pyrimidine derivatives, we found that the tumor uptake is related to the amount of radioactivity present in the blood. For both kinds of radiotracers, the initial radioactivity in the blood was similar high, but the clearance rate by 120 min was significantly different. The slow blood clearance rate of 2-[^18^F]fluoroethylaminopyrazolo[1,5-a]pyrimidine derivatives was along with higher uptake and longer retention in tumor. 2-[^18^F]fluoro-4-nitrobenzamide- pyrazolo[1,5-a] pyrimidine derivatives did the contrary. One of the disadvantages of this high circulating radioactivity in the blood would be not very clear visualization of tumor *in Vivo* using this kind of probe. The metabolic tissues such as liver and kidney showed initial high uptake but continued to clear out further with time. Meanwhile 2-[^18^F]fluoro-4-nitrobenzamidopyrazolo[1,5-a] pyrimidine derivatives washed out faster than 2-[^18^F]fluoroethylaminopyrazolo[1,5-a]pyrimidine derivatives did, which may derive from the distinct clearance rates from blood for both kinds of radiotracers.

### 2.7. MicroPET Imagings of [^18^F]3 in S180 Bearing Mouse

**Figure 5:**
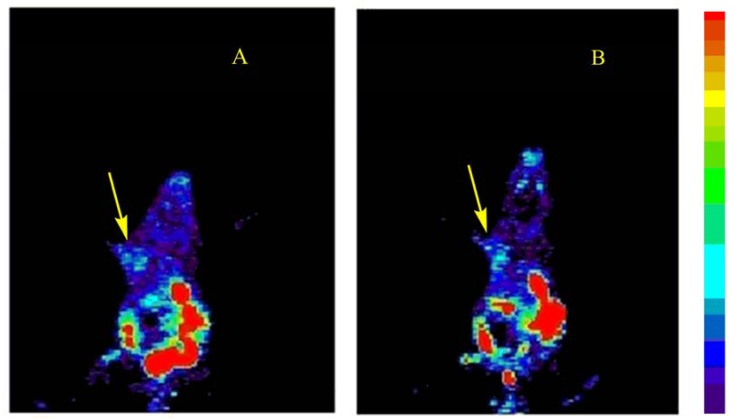
Coronal PET images of Kunmin mice with S180 tumor (arrow) in the left front flank at 30 min (**A**) and 60 min (**B**) post-injection of [^18^F]**3**.

Coronal microPET images of a mouse bearing S180 tumor at 30 and 60 min after tail vein injection of [^18^F]**3** are shown in Figure 5A,B, respectively. The tumor in the left forelimb was visible while no accumulation in the right forelimb was found. The regions of interest (ROI) ratio of [^18^F]**3** uptake for tumor site versus the corresponding non-tumor region (T/NT ratio) was 1.1 at 30 min and 1.5 at 60 min, suggesting that it would be able to accumulate in tumor for a relatively long time. The tumor-to-background contrast at 30 min p.i. was low, but it became a slightly better at 60 min p.i. These results were consistent with *ex vivo* biodistribution studies. Furthermore, [^18^F]**3** had low lipophilicity (logP = −0.04), which would expect to have less brain uptake [28]. By the comparison of the PET images at 30 min and 60min post-injection, the persistent retention of radiotracer in the brain was observed. 

## 3. Experimental

### 3.1. General

All chemicals were commercially obtained and used without further purification. Melting points were measured in capillary tubes using a RY-1 apparatus without correction. ^1^H-NMR spectra were recorded on a Bruker (400 MHz) spectrometer. ^13^C-NMR, ^19^F NMR spectra were recorded on a Bruker (100 MHz) spectrometer. Tetramethylsilane (TMS) was used as an internal standard for both spectra. Chemical shift data of the proton resonances were reported in parts per million (δ) relative to internal standard TMS (δ 0.0). Mass spectra were obtained using a Brucker Apex χ FTM instrument. Elemental analysis was performed on a Pekin-Elmer 240-C instrument. IR data were obtained on a Nicolet 360 Avatar infrared spectrophotometer. The [^18^F]fluoride used for radiosynthesis was produced by the ^18^O(p, n)^18^F nuclear reaction at Center of Xuanwu Hospital (Beijing, China). Semipreparative HPLC purification was carried out using a semipreparative reversed-phase Grace Alltima™ C_18_ Column (250 × 10 mm, particle size: 10 μm) under the indicated conditions. The Semipreparative HPLC system used was an Alltech system with Alltech HPLC pump Model 626, LINEAR UVIS-201, and BIOSCAN flow-counter. Reversed-phase extraction Sep-Pak C18 Plus cartridges (Waters) were activated with methanol and water before use. Compounds **9** and **12** shown in Scheme 1 and Scheme 2 were synthesized as described previously [27,29].

### 3.2. Synthesis

*(3-Cyano-7-(2-hydroxyethylamino)pyrazolo[1,5-a]pyrimidin-5-yl)methyl acetate *(**10**). To a solution of **9** (0.25 g, 1.0 mmol) in DMSO (7.5 mL) and water (5 mL) at room temperature were added acetic acid (0.15 g, 1.1 mmol) and NaOH (1.2 mg, 0.03 mmol). The reaction mixture was heated to reflux at 120 °C for 1.5 h and then cooled to room temperature. The solvent was removed under reduced pressure and the crude product was purified by column chromatography on silica gel eluted with 1:1 ethyl acetate/petroleum ether (60–90 °C) to afford **10** (0.16 g, 60%) as yellow solid, mp: 215 °C; IR (KBr, cm^−1^) υ: 3308.0, 3093.3, 2939.8, 2224.1, 1741.6, 1630.6, 1587.8, 1533.9, 1446.1, 1306.3, 1263.8, 1234.1, 1190.8, 1068.6; ^1^H-NMR (DMSO) δ 8.64 (s, 1H, Pyrazole-*H*), 6.50 (s, 1H, Pyrimidine-*H*), 5.09 (s, 2H, -C*H*_2_O-), 3.66–3.61 (m, 2H, OHC*H*_2_CH_2_-), 3.50 (dd, *J^1,2^* = 11.2 Hz, 5.56 Hz, 2H, -CH_2_C*H*_2_NH-), 2.17 (s, 3H, C*H*_3_CO-); ^13^C-NMR (DMSO, 100 MHz) δ 170.00, 159.86, 150.28, 148.07, 146.57, 114.11, 86.28, 78.47, 65.41, 59.31, 44.31, 20.59; MS (EI) *m/z*: 275.1, found: 276.3 [M+H]^+^; Anal. calcd for C_12_H_13_N_5_O_3_: C, 52.36; H, 4.76; N, 25.44; found: C, 52. 54; H, 4.77; N, 25.36.

*(3-Cyano-7-(2-(tosyloxy)ethylamino)pyrazolo[1,5-a]pyrimidin-5-yl)methyl acetate *(**11**). To a stirred solution of **10** (0.28 mg, 1 mmol) in CH_2_Cl_2_ (20 mL) cooled with ice bath (0 °C) was added to Et_3_N (0.21 mL, 1.5 mmol), p-toluenesulfonyl chloride (TsCl, 0.29 g, 1.5 mmol) and a catalytic amount of 4-dimethylaminopyridine (DMAP, 0.03 g, 0.2 mmol). After 30 minutes incubation on ice, the reaction was kept at room temperature overnight. The reaction was washed with water (1 × 12 mL), saturated sodium bicarbonate (1 × 10 mL), and brine (1 × 15 mL) successively. After drying over sodium sulfate, the volatile materials were evaporated under vacuum. The crude oily residue was further purified by column chromatography using petroleum ether (60–90 °C)/ethyl acetate (2:1) as eluant to give **11** (0.36 g, 85%) as white solid, mp: 150–152 °C. IR (KBr, cm^−1^) υ: 3329.0, 3101.8, 2221.8, 1735.6, 1628.7, 1586.4, 1451.2, 1361.7, 1320.8, 1231.0, 1188.3, 1175.7, 1032.8, 907.2, 807.5, 762.5, 665.0, 578.0, 550.8; ^1^H-NMR (DMSO) δ 8.63 (s, 1H, Pyrazole-*H*), 7.49 (d, *J *= 8.2 Hz, 2H, Ar-*H*), 6.98 (d, *J *= 8.1 Hz, 2H, Ar-*H*), 6.31 (s, 1H, Pyrimidine-*H*), 5.04 (s, 2H, -C*H*_2_O-), 4.28–4.30 (m, 2H, -CH_2_C*H*_2_NH-), 3.67–3.71 (m, 2H, OHC*H*_2_CH_2_-), 2.21 (s, 3H, C*H*_3_CO-), 2.19 (s, 3H, C*H*_3_-); ^13^C-NMR (DMSO, 100 MHz) δ 169.98, 159.78, 149.99, 147.01, 146.41, 144.39, 131.56, 129.20, 127.10, 114.09, 86.10, 78.68, 68.12, 65.27, 45.71, 20.84, 20.60; MS (EI) *m/z*: 429.1, found: 429.9 [M+H]^+^; Anal. calcd for C_19_H_19_N_5_O_5_S: C, 53.14; H, 4.46; N, 16.31; found: C, 53. 32; H, 4.47; N, 16.26.

*(3-Cyano-7-(2-fluoroethylamino)pyrazolo[1,5-a]pyrimidin-5-yl)methylacetate *([^19^F]**3**). Tetrabutyl- ammonium fluoride trihydrate (TBAF**^.^**3H_2_O, 0.63 g, 2 mmol) was dissolved in anhydrous CH_3_CN (1 mL) and heated rapidly at 120 °C under a nitrogen stream, followed by additional CH_3_CN (2 mL), the solvent was evaporated, this process was repeated three times. After evaporating most of the solvent, solution **11** (0.43 g, 1 mmol) in anhydrous CH_3_CN (3 mL) was added to the dry reagent. The reaction mixture was stirred at 75 °C for 2 h under a nitrogen steam and cooled to room temperature. The solvent was removed under reduced pressure. The residue was purified by silica gel chromatography using petroleum ether (60–90 °C)/ethyl acetate (1:1) as eluant to afford [^19^F]**3** (0.06 g, 20%) as a yellow solid. IR (KBr, cm^−1^) *υ*: 3379.9, 3093.8, 2940.1, 2225.0, 1730.3, 1627.3, 1597.0, 1548.3, 1442.7, 1379.8, 1258.0, 1062.2; ^19^F-NMR (CDCl_3_, 100 MHz) *δ* −223.00 (1F); ^1^H-NMR (CDCl_3_) *δ* 8.16 (s, 1H, Pyrazole-*H*), 6.73 (s, 1H, -N*H*-), 6.18 (s, 1H, Pyrimidine-*H*), 5.13 (s, 2H, -C*H*_2_O-), 4.68 (dt, *J^1,2^*= 46.9 Hz, 4.8 Hz, 2H, -C*H_2_*F), 3.73 (dq, *J^1,2^* = 25.9 Hz, 5.2 Hz, 2H, -C*H_2_*CH_2_F), 2.14 (s, 3H, C*H*_3_-); ^13^C-NMR (CDCl_3_, 100 MHz) *δ* 170.33, 160.12, 150.29, 148.00, 146.72, 114.22, 86.20, 82.05 (d, *J *= 173.0 Hz), 78.66, 65.37, 42.11 (d, *J *= 20.0 Hz), 20.61; MS (EI) *m/z*: 277.10, found: 277.9 [M]^+^; Anal. calcd for C_12_H_12_FN_5_O_2_: C, 51.98; H, 4.36; N, 25.26; found: C, 52.16; H, 4.37; N, 25.18.

*7-(2-Fluoroethylamino)-5-(hydroxymethyl)pyrazolo[1,5-a]pyrimidine-3-carbonitrile *([^19^F]**4**). The compound [^19^F]**3** (0.28 g, 1 mmol) was dissolved in CH_3_OH (10 mL) and a solution of NaOH (0.08 g, 2 mmol) in 2 mL H_2_O was added. The reaction mixture was heated at 80 °C for 20 min at which point TLC indicated the completion of the reaction. The yellow precipitate was formed after adjusting the pH to 7 with 1 N HCl aqueous solution and then filtered to afford [^19^F]**4** (0.2 g, 85%). IR (KBr, cm^−1^) *υ*: 3398.3, 2916.2, 2850.3, 2224.3, 1628.3, 1604.6, 1463.7, 1427.7, 1128.5, 1020.2, 624.7; ^19^F-NMR (DMSO) *δ* −221.66 (1F); ^1^H-NMR (DMSO) *δ *(ppm): 8.24 (s, 1H, Pyrazole-*H*), 6.75 (s, 1H, -N*H*-), 6.21 (s, 1H, Pyrimidine-*H*), 4.78 (d, 2H, *J *= 5.2 Hz, -C*H*_2_O-), 4.74 (dt, 2H, *J^1,2^*= 46.9 Hz, 4.8 Hz, -C*H_2_F), 3.84–3.74 (dm, 2H, J *= 25.9 Hz, -C*H_2_*CH_2_F), 3.34 (t, 1H, *J *= 5.2 Hz, -O*H*); ^13^C-NMR (DMSO, 100 MHz) *δ* 166.83, 150.35, 147.74, 146.55, 114.22, 85.41, 82.08 (d, 167 Hz), 78.04, 63.90, 42.97 (d, 22 Hz); MS (EI) *m/z*: 235.09, found: 235.1 [M]^+^; Anal. calcd for C_10_H_10_FN_5_O: C, 51.06; H, 4.29; N, 29.77; found: C, 51.24; H, 4.30; N, 29.68.

*(S)-Methyl 6-(3-cyano-5-methylpyrazolo[1,5-a]pyrimidin-7-ylamino)-2-(2,4-dinitrobenzamido)hexanoate *(**13**). To a stirred solution of L-lysine methyl ester hydrochloride (0.24 g, 1 mmol) in anhydrous CH_3_CN was successively added TEA (0.42 mL, 3 mmol) and the compound **12 **(0.19 g, 1 mmol), and the reaction mixture heated to reflux for 15 min. The resulting mixture was cooled and concentrated in vacuo. Thereafter, the residue was then dissolved in anhydrous CH_2_Cl_2_ (20 mL), cooled to 0 °C and treated successively with 2,4-dinitrobenzoic acid (0.21 g, 1 mmol), HOBt (0.12 g, 1 mmol) and DCC (0.23 g, 1 mmol) in CH_2_Cl_2_ (5 mL). The reaction mixture was stirred at 0 °C for 30 min and then at room temperature overnight. Following filtration the organic phase was washed with saturated sodium bicarbonate (1 × 10 mL), brine (1 × 15 mL), dried over sodium sulfate and evaporated to give a glassy residue, which was purified on silica gel eluted with petroleum ether (60–90 °C)/ethyl acetate (3:2) to give **13** (0.21 g, 42%) as yellow solid, mp: 157–158 °C; IR (KBr) *υ* (cm^−1^): 3372.7, 3295.3, 3099.5, 2941.2, 2863.5, 2222.9, 1752.4, 1638.0, 1612.0, 1588.8, 1540.7, 1465.3, 1449.1, 1351.7, 1303.1, 1230.0, 1230.0, 1172.5, 1158.4, 836.9, 739.5, 729.4, 671.4; ^1^H-NMR (CDCl_3_) *δ *8.91 (d, 1H, *J *= 2.1 Hz, Ar-*H*), 8.52 (dd, 1H, *J^1,2^*= 8.3, 2.2 Hz, Ar-*H*), 8.09 (s, 1H, Pyrazole-*H*), 7.78 (d, 1H, *J *= 8.3 Hz, Ar-*H*), 6.78 (d, 1H, *J *= 7.9 Hz, -N*H*(CO)), 6.42 (t, 1H, *J *= 5.7 Hz, -N*H*CH_2_), 6.04 (s, 1H, Pyrimidine-*H*), 4.91–4.86 (m, 1H, -(CO)C*H*CH_2_-), 3.83 (s, 3H, -OC*H_3_*), 3.49–3.44 (m, 2H, -HNC*H_2_*CH_2_), 2.56 (s, 3H, -C*H_3_*), 2.16–2.12 (m, 1H, -(CO)CHC*H*_2_-), 1.96–1.91 (m, 2H, -HNCH_2_C*H*_2_), 1.85–1.85 (m, 1H, -(CO)CHC*H*_2_-), 1.69–1.58 (m, 2H, -CHCH_2_C*H*_2_); ^13^C-NMR (CDCl_3_, 100 MHz) *δ *172.03, 164.06, 163.63, 150.38, 148.44, 146.74 (2C, Py-*C*, Ar-*C*), 146.03, 137.36, 130.30, 128.26, 120.21, 113.73, 88.17, 80.10, 52.97, 52.45, 41.98, 31.75, 28.14, 25.25, 22.27; MS (EI) *m/z*: 510.2, found: 510.5 [M]^+^; Anal. calcd for C_22_H_22_N_8_O_7_: C 51.76, H 4.34, N 21.95, found: C 51.94, H 4.35, N 21.88.

*(S)-Methyl 6-(3-cyano-5-methylpyrazolo[1,5-a]pyrimidin-7-ylamino)-2-(2-fluoro- 4-nitrobenzamido)hexanoate *([^19^F]**14**). Tetrabutylammonium fluoride trihydrate (TBAF**^.^**3H_2_O, 0.63 g, 2 mmol) was dissolved in anhydrous CH_3_CN (1 mL) and heated rapidly at 120 °C under a nitrogen stream, followed by additional CH_3_CN (2 mL), the solvent was evaporated, this process was repeated three times. After evaporating most of the solvent, solution **13** (0.51 g, 1 mmol) in anhydrous DMF (3 mL) was added to the dry reagent. The reaction mixture was stirred at 120 °C for 40 min under a nitrogen steam and cooled to room temperature, then diluted with H_2_O (20 mL), extracted with ethyl acetate (10 mL × 3). The combined organic phases were dried over Na_2_SO_4_, filtered and concentrated in vacuum. The residue was subjected to silica gel column chromatography (20% ethyl acetate in petroleum ether (60–90 °C)) to afford **14** (0.14 g, 29%) as yellow solid, mp: 62–63 °C; IR (KBr) *υ* (cm^−1^): 3384.1, 3104.7, 2953.2, 2866.3, 2223.7, 1742.5, 1670.1, 1625.8, 1586.1, 1532.3, 1457.3, 1419.8, 1353.1, 1306.5, 1226.0, 1192.1, 1154.8, 1092.1, 809.8, 738.0, 668.1; ^1^H-NMR (CDCl_3_) *δ* 8.30–8.27 (m, 1H, Ar-*H*), 8.15 (d, 1H, *J *= 7.5 Hz, Ar-*H*), 8.14 (s, 1H, Pyrazole-*H*), 8.05 (d, 1H, *J *= 11.0 Hz, Ar-*H*), 7.34 (m, 1H, -N*H*(CO)), 6.36 (m, 1H, -N*H*CH_2_), 6.02 (s, 1H, Pyrimidine-*H*), 4.92–4.87 (m, 1H, -(CO)C*H*CH_2_-), 3.82 (s, 3H, -OC*H_3_*), 3.46–3.41 (m, 2H, -HNC*H_2_*CH_2_), 2.58 (s, 3H, -C*H_3_*), 2.12–2.05 (m, 1H, -CH(CO)C*H*_2_), 1.96–1.87 (m, 2H, -HNCH_2_C*H*_2_), 1.87–1.79 (m, 1H, -(CO)CHC*H*_2_-), 1.63–1.56 (m, 2H, -CHCH_2_C*H*_2_); ^13^C-NMR (CDCl_3_, 100 MHz) *δ* 172.01, 163.51, 161.10 (d, *J *= 12.0 Hz, -HN*C*O), 159.83 (d, *J *= 236.0 Hz, Ar-*C*), 150.46, 150.44 (d, *J *= 4.0 Hz, Ar-*C*), 146.69, 146.08, 133.37 (d, *J *= 2.0 Hz, Ar-*C*), 126.10 (d, *J *= 12.0 Hz, Ar-*C*), 119.67 (d, *J *= 4.0 Hz, Ar-*C*), 113.52, 112.24 (d, *J *= 30.0 Hz, Ar-*C*), 87.99, 80.49, 52.82, 52.52, 41.94, 32.23, 28.14, 25.28, 22.58; ^19^F-NMR (CDCl_3_) *δ *−109.18 (1F); MS (EI) *m/z*: 483.2, found: 483.2 [M]^+^; Anal. calcd for C_22_H_22_FN_7_O_5_: C 54.66, H 4.59, N 20.28, found: C 54.74, H 4.60, N 20.22.

*(S)-6-(3-Cyano-5-methylpyrazolo[1,5-a]pyrimidin-7-ylamino)-2-(2-fluoro-4-nitrobenzamido)hexanoic acid* ([^19^F]**5**). To a solution of [^19^F]**14** (48 mg, 0.1 mmol) in CH_3_OH (3 mL) cooled to 0 °C was added LiOH·H_2_O (21 mg, 0.5 mmol) in H_2_O (0.6 mL). After 30 min, the cold bath was removed, and the reaction was continued at ambient temperature overnight. The reaction mixture was concentrated in vacuo and the pH was adjusted to 2 with 35% HCl at which point a yellow precipitate was formed. The precipitate was filtered, washed thoroughly with water, and then crystallized from methanol/ether to produce [^19^F]**5** (0.45 mg, 95%) as yellow solid, mp: 224–226 °C; IR (KBr) *υ* (cm^−1^): 3394.4, 3103.7, 2934.8, 2226.3, 1656.4, 1619.4, 1581.6, 1531.8, 1417.1, 1353.5, 1311.0, 1192.7, 1014.2, 810.1, 739.5, 650.1; ^1^H-NMR (DMSO) *δ *8.54 (s, 1H, Pyrazole-*H*), 8.39 (m, 1H, -N*H*(CO)), 8.28 (t, 1H, *J *= 5.8 Hz, -N*H*CH_2_), 8.18 (dd, 1H, *J *= 10.5, 2.0 Hz, Ar-*H*), 8.13 (dd, 1H, *J *= 8.6, 2.0 Hz, Ar-*H*), 7.99–7.95 (m, 1H, Ar-*H*), 6.39 (s, 1H, Pyrimidine-*H*), 3.99–3.95 (m, 1H, -C*H*(CO)CH_2_-), 3.36–3.33 (m, 2H, -NHC*H_2_*CH_2_), 2.43 (s, 3H, -C*H_3_*), 1.88–1.84 (m, 1H, -CH(CO)C*H*_2_-), 1.73–1.70 (m, 1H, -CH(CO)C*H*_2_), 1.61–1.59 (m, 2H, -NHCH_2_C*H*_2_), 1.39–1.34 (m, 2H, -CHCH_2_C*H*_2_); ^13^C-NMR (DMSO, 100 MHz) *δ *173.64, 162.62, 160.04 (d, *J *= 9.0 Hz, -NH*C*O), 158.70 (d, *J *= 251.0 Hz, Ar-*C*), 150.50, 149.01 (d, *J *= 10.0 Hz, Ar-*C*), 146.82, 145.99, 131.66 (d, *J *= 3.0 Hz, Ar-*C*), 129.21 (d, *J *= 15.0 Hz, Ar-*C*), 119.49 (d, *J *= 3.0 Hz, Ar-*C*), 114.39, 111.98 (d, *J *= 29.0 Hz, Ar-*C*), 88.22, 77.61, 54.68, 41.29, 31.76, 28.01, 24.39, 22.18; ^19^F-NMR (DMSO) *δ* −110.47 (1F); MS (EI) *m/z*: 469.2, found: 470.4 [M+H]^+^; Anal. calcd for C_21_H_20_FN_7_O_5_: C 53.73, H 4.29, N 20.89, found: C 53.92, H 4.30, N 20.83.

*(3-Cyano-7-(2-[^18^F]fluoroethylamino)pyrazolo[1,5-a]pyrimidin-5-yl)methyl acetate *([^18^F]**3**), *7-(2-[^18^F]fluoroethylamino)-5-(hydroxymethyl)pyrazolo[1,5-a]pyrimidine- 3-carbonitrile* ([^18^F]**4**) *and (S)-6-(3-cyano-5-methylpyrazolo[1,5-a]pyrimidin- 7- ylamino)- 2-(2-[^18^F]fluoro-4-nitrobenzamido)hexanoic acid* ([^18^F]**5**). [^18^F]Fluoride was generated from a [^18^O(p,n)^18^F] nuclear reaction by irradiation on 97% enriched H_2_^18^O (Beijing PET Center of Xuanwu Hospital). [^18^F]fluoride was trapped on an anion exchange cartridge and directly eluted into a 10 mL sealed reaction vessel using a solution of potassium carbonate (3–5 mg) and Kryptofix 2.2.2 (15–20 mg) in 1.5 mL acetonitrile/water (3:1). The solvents were removed under a stream of nitrogen at 110 °C. Subsequently, 0.8–1.0 mL of dry acetonitrile was added three times and evaporated to dryness. To the obtained dry [^18^F] K2.2.2/KF complex, 11 (3 mg, 7 μmol) in dry CH_3_CN (0.5 mL) was added. The solution was heated at 55–80 °C for 40 min and diluted with H_2_O (10 mL) and loaded onto an activated C18 Sep-Pak cartridge. The Sep-Pak cartridge was washed with H_2_O (10mL) to remove unlabeled ^18^F and the [^18^F]**3** was then eluted with 2 mL of CH_3_CN, which was injected into a semi-preparative HPLC performed on Grace Alltima™ C18 Column (250 × 10 mm, particle size: 10 μm) isocratically eluted with CH_3_CN/H_2_O = 40:60 at a flow rate of 3 mL/min. The HPLC solvents were removed under reduced pressure to give [^18^F]**3**. Hydrolysis was rapid following the addition of LiOH (10 mg) in MeOH (2 mL) at 80 °C for 20 min and then neutralized with 1 N HCl. The solvent was evaporated *in vacuo* to give [^18^F]**4**. 

To synthesize [^18^F]**5**, **13** (5 mg, 10 μmol) in dry DMF (0.5 mL) was added to the vial containing dry [^18^F] K2.2.2/KF complex and heated at 110 °C for 40 min, diluted with H_2_O (10 mL) and loaded onto an activated C18 Sep-Pak cartridge. The Sep-Pak cartridge was washed with H_2_O (10 mL) to remove unlabeled ^18^F and the [^18^F]**14** was then eluted with 2 mL of CH_3_CN, which was injected into a semi-preparative HPLC performed on Grace Alltima™ C18 Column (250 × 10 mm, particle size: 10 μm) isocratically eluted with CH_3_CN/H_2_O = 40:60 at a flow rate of 3 mL/min. The elution was collected after purification and the solvents were removed under reduced pressure to provide [^18^F]**14**, subsequently hydrolyzed using LiOH (10 mg) in MeOH (2 mL) at 80 °C for 20 min and then adjusted pH to 7 with 1 N HCl. The solvent was evaporated *in vacuo* to give [^18^F]**5**. The product was dissolved in certain amount of phosphate-buffered saline (pH = 7.4) (370 kBq/0.1 mL) for further use.

### 3.3. Octanol/Water Partition Coefficient

The partition coefficient was measured as previously described [30]. Each HPLC-purified radiotracer was added to a tube containing 0.5 mL of n-octanol and 0.5 mL of phosphate-buffered saline (pH 7.4). Each phase had been presaturated with the opposite phase. The mixed solution vortexed for 1 min and centrifuged at 15,000 rpm for 2 min. Aliquots of both phases were transferred in triplicate to counting tubes and assayed in a γ counter. The partition coefficient was calculated by dividing the radioactivity of the octanol layer with that of the water layer. This measurement was repeated three times.

### 3.4. Quality Control of Purified Radiotracers

Quality control for [^18^F]**3**, [^18^F]**4** and [^18^F]**5** using authentic [^19^F]**3**, [^19^F]**4** or [^19^F]**5** as a reference was done by Radio-HPLC on Grace Alltima™ C18 Column (250 × 10 mm, particle size: 10 μm) isocratically eluted with CH_3_CN/H_2_O = 40:60 at a flow rate of 3 mL/min. Using this system, specific activity was calculated by the UV peak area of the purified radiotracers as compared with standard curves of a reference nonradioactive compound.

### 3.5. Stability Study

Each HPLC-purified radiotracer (3.7 MBq) was mixed with phosphate-buffered saline (pH 7.4) at 37 °C for a period up to 2 h and was analyzed by radio-HPLC. The stability of each HPLC-purified radiotracer in mouse plasma was determined by incubating 0.1 mL of radiotracer (3.7 MBq) in the solution of 0.5 mL murine plasma at 37 °C for 1 h and 2 h. Plasma proteins were precipitated by adding acetonitrile and removed by centrifugation. The supernatant part was injected into radio-HPLC on Grace Alltima™ C18 Column (250 × 10 mm, particle size: 10 μm) isocratically eluted with CH_3_CN/H_2_O = 40:60 at a flow rate of 3 mL/min using the corresponding non-radioactive [^19^F]**3**, [^19^F]**4** and [^19^F]**5** as references to determine the stability of each compound.

### 3.6. Cellular Accumulation Studies

Cellular accumulation studies were performed with S180 tumor cells, which were suspended in fresh DMEM medium supplemented with 10% fetal bovine serum at a cell concentration of 2 × 10^6^ cells/mL and incubated at 37 or 4 °C with gentle stirring under an atmosphere of 95% air plus 5% carbon dioxide. The cells were incubated together with each purified radiotracer (0.25 MBq in 0.15 mL of cell culture medium) at 37 or 4 °C. At 5, 15, 30, 60 and 120 min, 200 μL samples of the suspension were transferred to microfuge tubes and centrifuged at 1500 rpm for 5 min. A 180-μL sample of each supernatant was removed for counting, and the left sample containing cells and 20 μL medium was also counted. Percent cell uptake is calculated as [residue counts-(supernatant counts/9)]/(residue counts + supernatant counts) × 100%. The data are expressed as mean ± S.D. (n = 5). During all the experiments, cell viability was always greater than 90%. The method is validated and the relevant references have been listed [25,31].

### 3.7. Biodistribution and MicroPET Imaging Studies in S180 Tumor Bearing Mice

All the animal studies were carried out in compliance with relevant national laws relating to the conduct of animal experimentation. Uptake of [^18^F]**3**, [^18^F]**4** and [^18^F]**5** in Kunming Mice bearing S180 tumor models were performed according to published methods [27]. Briefly, S180 tumor cells were trypsinized, counted, and suspended in a solution containing DMEM and 10% fetal bovine serum. Kunming Mice were inoculated subcutaneously into the left forelimb with 5 × 10^6^ tumor cells. Studies of tumor uptake were conducted when the tumors reached a size of 0.5–0.8 cm in diameter. The tumor-bearing mice were subjected to *in Vivo* biodistribution and imaging studies. 

Tissue biodistribution studies were conducted by administering, via the tail vein, a bolus injection of 370 kBq/mouse in a constant volume of 0.1 mL phosphate-buffered saline solution (pH = 7.4). The animals were sacrificed at 5, 15, 30, 60 and 120 min post injection. The tissues and organs of interest were immediately collected, weighed and measured for ^18^F radioactivity in a γ counter. Values are expressed as mean ± SD (n = 4). 

For small-animal PET (Siemens Inveon dedicated microPET), the mouse was injected intravenously with approximately 3.7 MBq of [^18^F]**3** in saline, anesthetized with 1.5% isoflurane and fixed near the center of microPET scanner. Static scans (for 30 and 60 min, 10-min scans) were obtained, the data were histogrammed into 25 consecutive frames and the images were reconstructed by a three dimensional ordered subsets expectation maximum (OSEM) algorithm. The manually selected region of interest (ROI) was applied to each image to estimate the counts present in the tumor and the corresponding nontumor region by Amide’s a Medical Image Data Examiner (AMIDE) software.

### 3.8. Statistical Analysis

Data are expressed as the mean ± S.D. Mean values were compared using Student's *t* test. Differences were considered statistically significant for *P* ≤ 0.05.

## 4. Conclusions

In conclusion, on the basis of our previous studies, we designed, synthesized and evaluated the biological characteristics of three novel ^18^F-labeled pyrazolo[1,5-a]pyrimidine derivatives: [^18^F]**3**, [^18^F]**4** and [^18^F]**5**. During *in Vivo* animal studies, we observed that biodistribution studies revealed generally similar uptake patterns of [^18^F]**1**, [^18^F]**3** and [^18^F]**4** for most organs and the tumor, while [^18^F]**5** showed similar distribution patterns as for [^18^F]**2** in the most organs and the tumor. PET images indicated that the persistent tumor uptake of [^18^F]**3 **was visible. By comparison among the five compounds, 2-[^18^F]fluoroethylaminopyrazolo[1,5-a]pyrimidine derivatives: [^18^F]**1**, [^18^F]**3** and [^18^F]**4** offered significant advantages over 2-[^18^F]fluoro-4-nitrobenzamidopyrazolo[1,5-a]pyrimidine derivatives: [^18^F]**2** and [^18^F]**5** with regard to notably and persistent tumor uptake. These results implied that tosylate precursor for F-18 labeled pyrazolo[1,5-a] pyrimidine core structure was more efficient than a 2,4-dinitrobenzamide precursor and the fact that neither 2-[^18^F]fluoroethylaminopyrazolo[1,5-a]pyrimidines ([^18^F]**1**, [^18^F]**3** and [^18^F]**4**) nor 2-[^18^F]fluoro-4-nitrobenzamidopyrazolo[1,5-a] pyrimidines ([^18^F]**2** and [^18^F]**5**) showed any relationship between their lipophilicity and biodistribution behavior. These results are interesting enough to be merit more effort in further research.

## Supplementary Materials

Supplementary materials can be accessed at: http://www.mdpi.com/1420-3049/17/4/3774/s1.
