# Supplementary material for: 18F-labeled Pyrazolo[1,5-a]pyrimidine Derivatives: Synthesis from 2,4-Dinitrobenzamide and Tosylate Precursors and Comparative Biological Evaluation for Tumor Imaging with Positron Emission Tomography
Source: Molecules. 2012 Mar 27;17(4):3774–93. doi: 10.3390/molecules17043774 (PMC6268720; doi:10.3390/molecules17043774)

Figure S1. The HPLC chromatogram of [^{19}F]3 (**A**) and the radiochromatogram of [^{18}F]3 (**B**) The retention time of [^{19}F]3 (10.9 min) was measured with a UV detector while the retention time of [^{18}F]3 (11.3 min) was measured radiometrically.

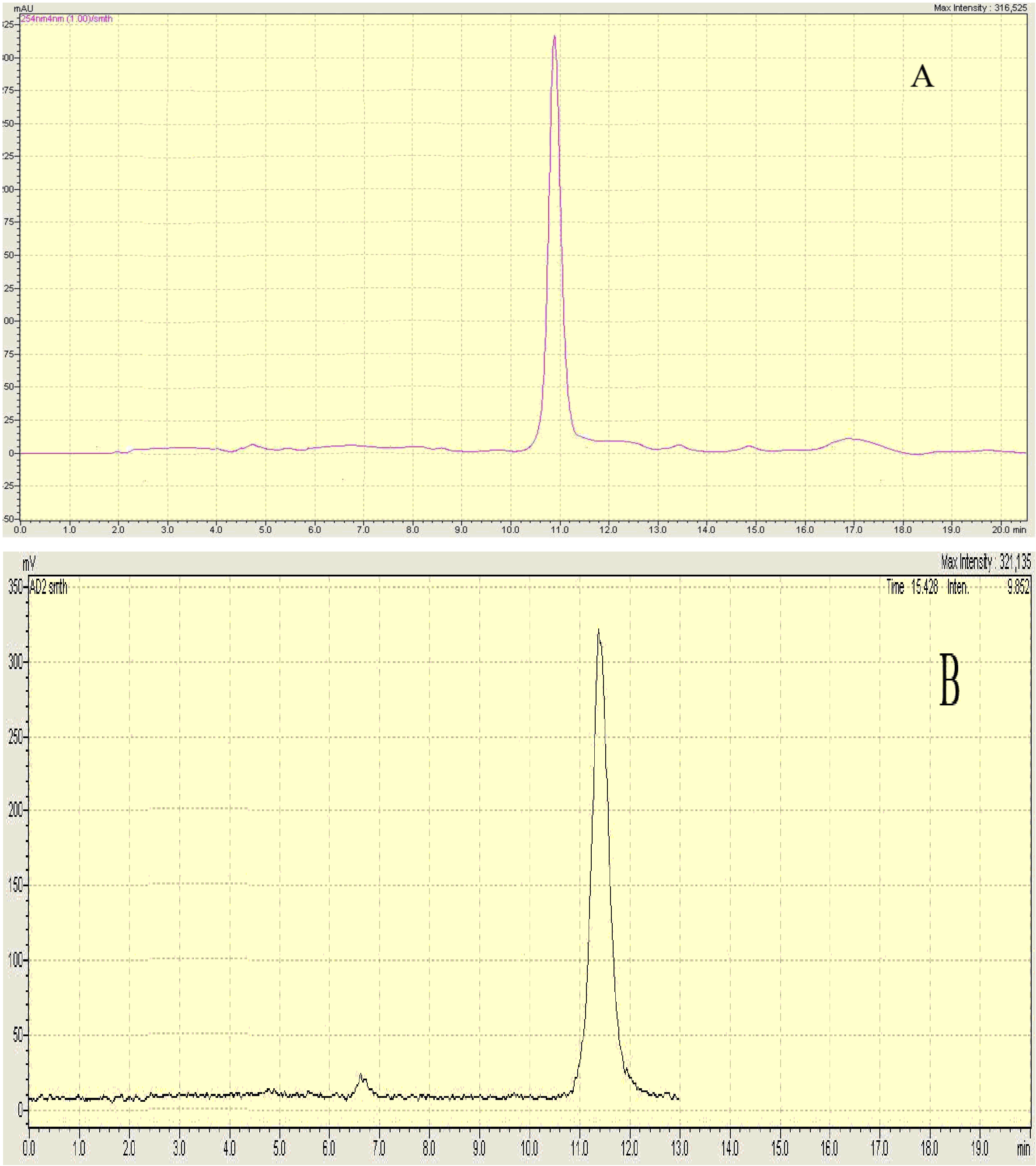

Figure S2. The HPLC chromatogram of [^{19}F]4 (**A**) and the radiochromatogram of [^{18}F]4 (**B**). The retention time of [^{19}F]4 (5.8 min) was measured with a UV detector while the retention time of [^{18}F]4 (6.5 min) was measured radiometrically.

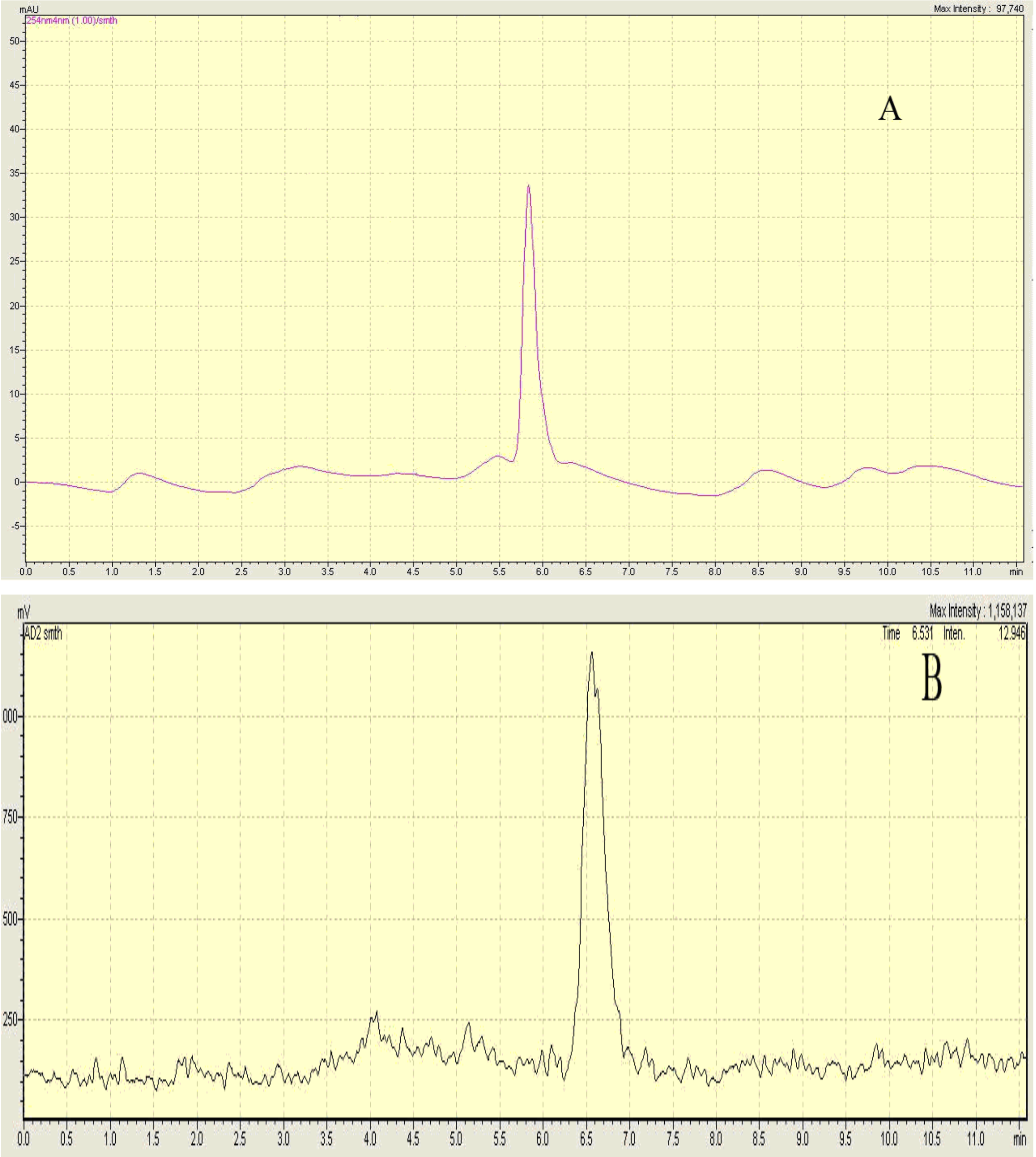

Figure S3. The HPLC chromatogram of [^{19}F]5 (**A**) and the radiochromatogram of [^{18}F]5 (**B**). The retention time of [^{19}F]5 (2.15 min) was measured with a UV detector while the retention time of [^{18}F]5 (2.34 min) was measured radiometrically.

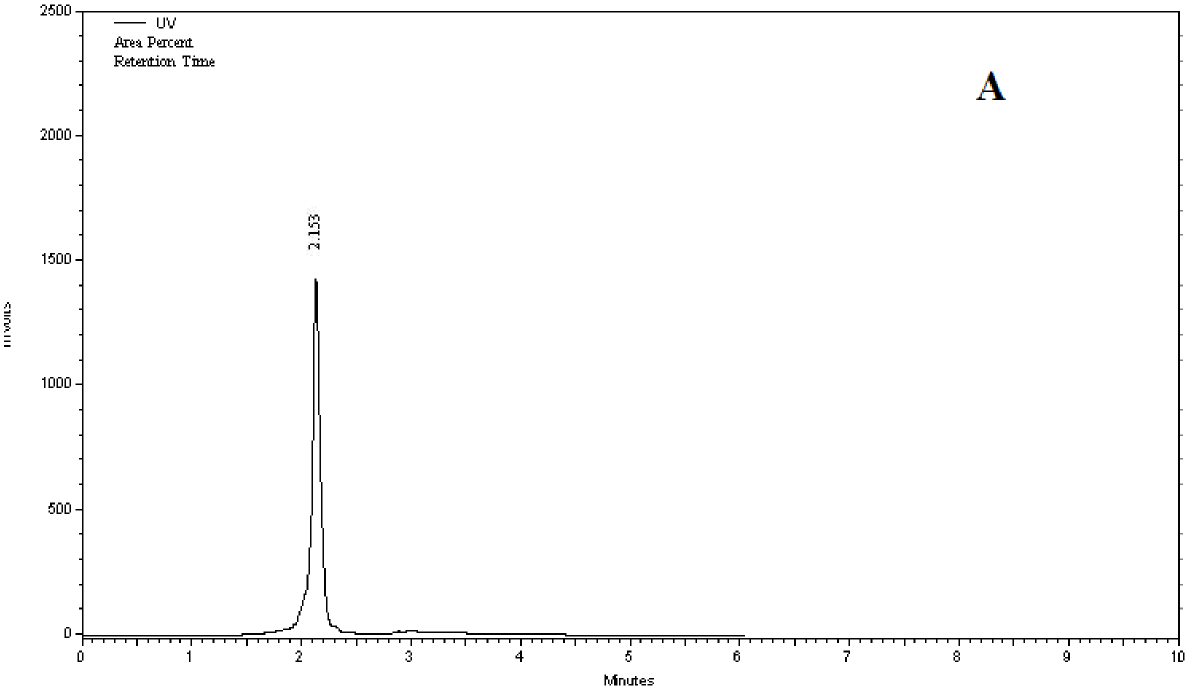

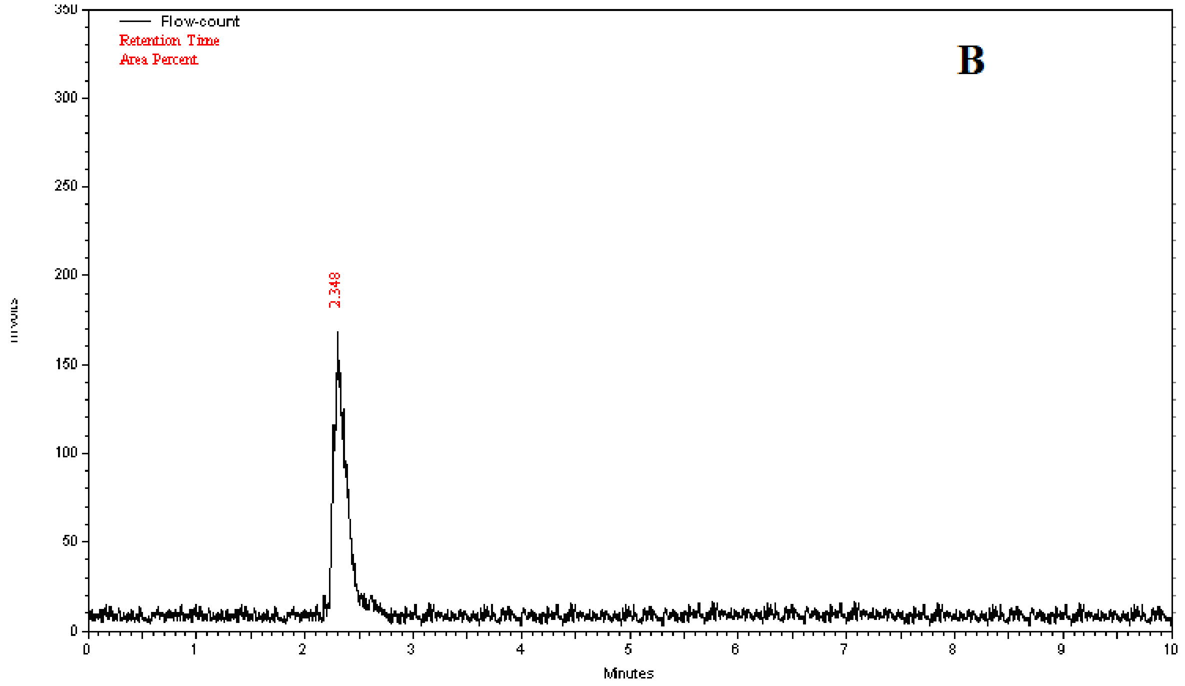

Supplement: Supplementary file 1 [file molecules-17-03774-s001.pdf]
